# Gular Cutaneous Fibrosarcoma and Anatomophysiological Considerations for Anaesthesia in a Saddled Bichir, *Polypterus endlicheri endlicheri*

**DOI:** 10.1155/2022/2199005

**Published:** 2022-01-17

**Authors:** Ayanna Carla N. Phillips Savage, Karelma Frontera-Acevedo

**Affiliations:** ^1^Department of Clinical Veterinary Sciences, School of Veterinary Medicine, Faculty of Medical Sciences, The University of the West Indies, St. Augustine, Trinidad and Tobago W.I.; ^2^Department of Basic Veterinary Sciences, School of Veterinary Medicine, Faculty of Medical Sciences, The University of the West Indies, St. Augustine, Trinidad and Tobago W.I.

## Abstract

Bichirs (*Polypteru*s spp.) have frequently been studied with regard to comparative and developmental anatomy; however, very little information has been published regarding diseases, aging changes, and medical and surgical management in these species. Neoplasia represents one such example of conditions for which a dearth of information in these species exists. There has been increasing recognition of various types of neoplasms, including cutaneous tumors, particularly in ornamental fish; some of which may be related to environmental or to infectious causes. When excision of such tumors is indicated, surgical anaesthesia is required. However, special considerations may be warranted when employing immersion anaesthesia protocols in facultative air-breathing fish that can utilise the lungs for respiration. This anatomophysiological feature of *Polypterus* spp. may not only influence induction and maintenance of surgical anaesthesia but may theoretically have implications for drowning. Herein, we describe the management of a case of a rapidly growing gular neoplasm in a juvenile saddled bichir and considerations for surgical anaesthesia in this “lunged” species. Induction and maintenance of surgical anaesthesia using tricaine methanesulfonate (MS-222) in this species were found to be effective at significantly lower concentrations than standard recommended doses. Histopathological analysis identified the mass as a fibrosarcoma. To the best of our knowledge, this is the first report of a cutaneous fibrosarcoma in a bichir, representing the first report of neoplasia in *Polypteru*s spp. and the first description of surgical anaesthesia in this amphibious fish.

## 1. Introduction

Bichirs (*Polypterus* spp.) are primitive ray-finned, freshwater fish of African origin [1]. They are among the few fish species that have lungs and can breathe air, and thus, it is their evolutionary and developmental anatomy that has spawned great research interest [1, 2]. Polypterids exhibit bimodal respiration, having the ability to achieve gaseous exchange both aquatically and aerially [3, 4]. Paired functional tubes, known as spiracles, extend from the dorsal surface of the skull into the buccopharyngeal chamber and lead to ventrally paired, asymmetrical, sac-like lungs via a glottal valve [3–8]. The walls of the lungs consist of tissue layers that differ in their functionality [6]. The surface epithelium lining the pulmonary lumen is composed of pneumocytes I, pneumocytes II containing lamellar bodies (presumed to be associated with surfactant production), mucous cells, and ciliated cells [6]. Just beneath pneumocytes I is a dense capillary network; the thin continuous endothelium of which, along with pneumocytes I, forms the very thin blood-air barrier [6]. Beneath the region of gas-exchanging tissue is a layer of connective tissue consisting of collagen and special elastic fibers [6]. The serosal surface of the lungs consists of a narrow inner zone of smooth muscle cells and a broader outer zone of cross-striated muscle cells [6]. Pulmonary innervation is effected by myelinated and nonmyelinated nerve fibers [6]. Pulmonary circulation and pulmonary gas-exchanging tissue morphology are notably very similar to those of tetrapod vertebrates [3, 5, 6, 8].

When the concentration of dissolved oxygen in the water is suboptimal, polypterids preferentially utilise their spiracles and lungs, instead of their gills, for respiration via a process known as recoil aspiration [5, 9]. During recoil aspiration, muscles within the lung wall contract, expelling air from the lungs into the buccopharyngeal chamber and out through the opercula [9]. The negative pressure thus created within the body cavity causes the body wall to collapse inward [9]. When the glottal valve opens, the body wall recoils and the subambient pressure aspirates air into the lungs [9]. This atypical respiratory anatomophysiological feature should therefore be considered, particularly in the surgical management of polypterid patients.

*Polypterus* spp. are known to live for over 30 years in captivity [10]. Despite this considerable life span, very little has been published regarding any diseases or conditions present in these species, with the exception of some parasites [11, 12]. Numerous species of fish are known to be particularly long-lived, with wild and captive species being known to live for many decades to over a century [13, 14]. Environmental quality and, in the case of captive animals, husbandry practices influence longevity, with water quality having a critical influence on physiological parameters including immune function in fish [15]. Immune suppression, regardless of its underlying cause, increases susceptibility to infectious and noninfectious diseases [15, 16]. The pathogenesis of neoplasia in fish requires continued study, as more neoplasms are being identified across species as more fish are being kept in captivity over extended periods of time. While the causes of most neoplasms in fish have not been definitively identified, known causes include inherited genetic mutations, acquired mutations secondary to exposure to environmental contaminants such as carcinogenic chemicals, exposure to infectious agents, most notably retroviruses and herpesviruses, and possible linkages to ultraviolet B (UVB) radiation [17–20].

Round cell, epithelial and mesenchymal tumors have all been described in fish, with ectoderm- and endoderm-derived epithelial tumors being the most commonly occurring and the skin being the most commonly observed site of occurrence [19, 21, 22]. While cutaneous fibromas are not uncommon in fish, fibrosarcomas, which are common skin tumors in domestic animals like dogs and cats, are reportedly less common in fish, having been documented in the oral cavity, operculum, and caudal peduncle of a catfish (*Hemiarius dioctes*), the skin of the lateral body wall of a goldfish (*Carassius auratus*) and a hooknose (*Agonus cataphractus*), and on the flank near the dorsal fin of a perch (*Perca flavescens*) [13, 17, 19, 22–28]. Herein, we report on surgical anaesthetic considerations and the clinicopathological features of a cutaneous fibrosarcoma in the gular region of a saddled bichir (*Polypterus endlicheri endlicheri*) in Trinidad and Tobago.

## 2. Case Description

### 2.1. Background

**Figure 1:**
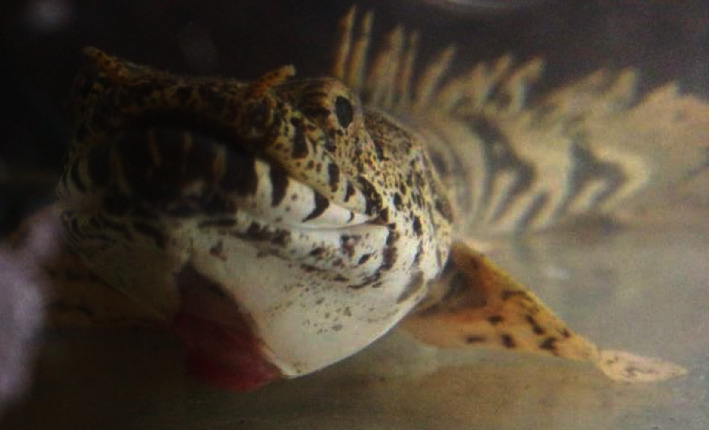
Mass in the gular region of a saddled bichir (*Polypterus endlicheri endlicheri*) on first observation.

An approximately 2.5-year-old saddled bichir was presented at the Aquatic Animal Health Unit at the University of the West Indies, School of Veterinary Medicine in Trinidad and Tobago. The presenting complaint was that the owner suspected the animal suffered a laceration to the throat just over 1 week prior and pink tissue was protruding ventrally (Figure 1). The owner reported that in the days since the laceration was first observed, the size of the protruding tissue appeared to be increasing. The animal became anorectic approximately 2 days before presentation but was still alert and active.

### 2.2. Clinical Evaluation

**Figure 2:**
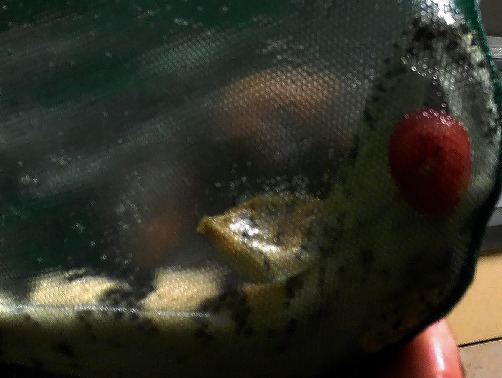
Firm, spherical, centrally ulcerated mass in the gular region of a saddled bichir (*Polypterus endlicheri endlicheri*) on clinical presentation, 1 week after first observation. The mass emerges from tissue deep to the axial margins of gular plates, pushing the plate margins abaxially.

On physical examination, the animal was in good body condition, weighed 151.91 grams, and measured approximately 32.5 cm. There was no evidence of a ventral laceration, but rather the gular plates were being separated and pushed abaxially by a prominent, moderately firm, broad-based, roughly spherical mass measuring approximately 2 cm in diameter. The mass was hyperaemic, with mildly friable areas that were prone to haemorrhage. The ventral aspect of the mass was smooth but ulcerated centrally, with a white, soft concavity (Figure 2). Skin scrape and fin clip assessments were unremarkable.

Based on the location of the mass and the report of its rapid growth, the main differentials were thyroid hyperplasia (goiter) or a malignant thyroid neoplasm. A fine needle aspirate submitted for cytological analysis revealed evidence of necrosis, but was nondiagnostic. A second aspirate taken 1 week later yielded the same results, and biopsy with histopathological examination was recommended for a definitive diagnosis. A decision was made to surgically excise the rapidly growing mass for histological assessment. For financial reasons, the owner opted to donate the animal to the UWI-SVM for teaching purposes prior to scheduling the surgery.

### 2.3. Surgical Anaesthesia and Postoperative Recovery

The animal was premedicated with intramuscular administration of meloxicam (Metacam®, Boehringer Ingelheim International, Binger Strasse, Germany) at 0.2 mg/kg for pain management and oxytetracycline hydrochloride (Phenix®, Kela N.V., Sint-Lenaartseweg 48, Belgium) at 25 mg/kg. The animal was immersed in a bath containing tricaine methanesulfonate (Tricaine-S, MS-222) (Syndel Laboratories, Western Chemical and Aquatic Life Sciences, Ferndale, Washington USA) at a dose of 50 ppm buffered at a 1 : 2 ratio with sodium bicarbonate. This concentration proved adequate to induce anaesthesia (loss of righting reflex, reduction in opercular beats, loss of tail and fin motion, and loss of response to external stimuli) in this animal within 2 minutes. The animal was quickly removed from the bath and placed on a recirculating fish anaesthesia delivery system. Continuous delivery of well-aerated (7.5 ppm-8.0 ppm dissolved oxygen at 27°C) buffered anaesthesia water containing MS-222 at a concentration of 35 ppm to the animal's gills was found to be adequate for maintenance of anaesthesia in this patient. The base of the mass was infiltrated with lidocaine hydrochloride (Hospira, Inc. Lake Forest, Illinois USA) at 1.5 mg/kg. On surgical excision of the mass, the defect in the body wall precluded primary closure, therefore favouring postoperative management that would allow for healing by secondary intention. The site was therefore packed with 1% silver sulfadiazine cream (Flamazine™, Smith & Nephew Pty. Ltd., Victoria, Australia), and a thin layer of 0.2% benzalkonium chloride liquid bandage (New Skin® Liquid Bandage, Advantice Health, LLC, New Jersey USA) was applied and allowed to dry thoroughly. The animal was immersed in well-aerated freshwater while being supported near the surface. Recovery was smooth and occurred within 5 minutes. The animal was returned to an aquarium in an oxytetracycline bath at a concentration of 50 mg/L, and environment was kept dark to prevent photoinduced deterioration of the oxytetracycline. The topical antibiotic application was to be continued daily until the defect had healed, and the bath treatment was to be maintained for 10 days with a 50-75% daily water change. While the animal survived the surgical procedure and initially appeared to be doing well, it was found dead two days later, presumably due to osmolar disruption secondary to compromise of the cutaneous integrity upon tumor excision since the nature of the site prohibited effective surgical wound closure. Unfortunately, due to inappropriate carcass storage by laboratory personnel, a full necropsy could not be performed due to loss of diagnostic quality of the specimen.

### 2.4. Gross Description

**Figure 3:**
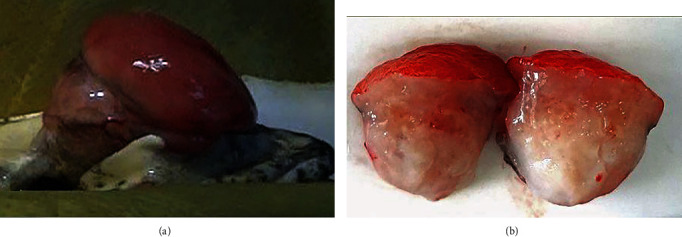
Mass in the gular region of a saddled bichir (*Polypterus endlicheri endlicheri*). (a) Gular mass immediately prior to surgical excision. The mass is seen to be emerging from tissue deep to the axial margins of gular plates, pushing the plate margins abaxially. (b) Cut surface of excised gular mass revealing its moist appearance, white colour and moderately firm consistency.

The diameter of the mass had increased from its original measurement of approximately 2.0 cm to approximately 2.5 cm, and its depth was roughly equivalent to the diameter (Figure 3(a)). On cut surface, the mass was moist, white, and moderately firm in consistency (Figure 3(b)). The sections were fixed in 10% neutral buffered formalin and submitted for histopathological assessment.

### 2.5. Histopathology

**Figure 4:**
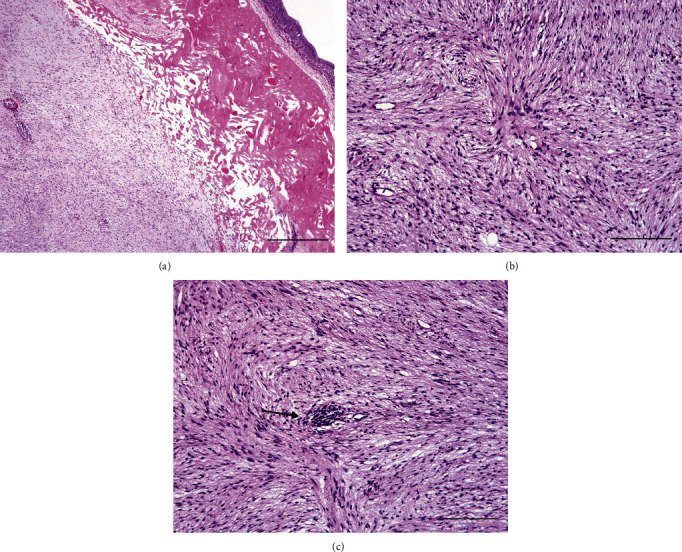
Histological features of mass excised from the gular region of a saddled bichir (*Polypterus endlicheri endlicheri*). (a) Unencapsulated mass in the deep dermis with streams and bundles. Hematoxylin and eosin, bar = 500 *μ*m. (b) The mass is composed of streams and bundles of spindled cells. Hematoxylin and eosin, bar = 100 *μ*m. (c) Rare, small lymphocytic clusters are present around some vessels (arrow). Hematoxylin and eosin, bar = 100 *μ*m.

The mass was embedded in paraffin, sectioned at 5 *μ*m thickness, and stained with hematoxylin and eosin (H&E), according to standard laboratory techniques [29]. The sections were examined using an Olympus BX 41 light microscope. The deep dermis and subcutaneous area were expanded by a nonencapsulated but well-circumscribed, highly cellular mass composed of streams and interlacing bundles of spindle-shaped cells supported by vascular stroma (Figures 4(a) and 4(b)) that rarely infiltrated to the surrounding dermis. The cells contained scant to moderate eosinophilic cytoplasm and a round to oval nucleus with smooth to finely stippled chromatin pattern with occasional multiple small nucleoli. There was mild to moderate anisocytosis and anisokaryosis, and no mitotic figures were observed in 2.37 mm^2^. Occasional small clusters of lymphocytes and plasma cells were noted throughout the mass (Figure 4(c)). Only small amounts of collagen were present in this mass, especially when compared with the surrounding tissue. One region appeared to be ulcerated with some granulation tissue in the surface. A well-differentiated fibrosarcoma was diagnosed based on histological findings. The cytological findings of necrosis could correspond to the superficial ulcerated area.

The case timeline is presented in Table 1.

## 3. Discussion

This case presented two uncommon clinical issues in teleost patients. Firstly, this was a case of an atypical gular tumor in a teleost. The most described mass in the gular region of teleost fish is associated with thyroid hyperplasia (goiter) [30–32]. Fibrosarcomas are common tumors in domestic animal species and can range from well-differentiated sarcomas with low amount of mitotic activity and simple spindle cells to more aggressive, multinucleated tumors with high mitotic activity [33]. While these tumors have been diagnosed in several fish species, their occurrence is comparatively less common in fish. Further, a search of the literature revealed no record of this or any other tumor or disease process in bichirs. As described earlier, the aetiology of this malignancy in fish has most often been linked to viral, particularly retroviral, infections or exposure to chemical pollutants [17, 21, 24, 25, 34]. The relatively young age of the bichir in this case suggests that it is unlikely to have been spontaneous, but perhaps associated with a viral, chemical, or inflammatory aetiology.

A review of the history in this case revealed that the animal was housed in an approximately 300 US gallon aquarium with 7 other fish; the species and sizes of which were not indicated. Filtration was effected via a sponge filter, a moving bed filter, and an ultraviolet (UV) sterilizer. The owner indicated that pH and total ammonia were tested monthly and were usually approximately 7 and 0 ppm, respectively. The animals were fed live blackworms (*Lumbriculus variegatus*), raw beef heart, and a commercial carnivore pellet. While the tank volume and selected water quality parameter readings provided appeared adequate for a single bichir, the absence of other environmental factors that could have served as predisposing stressors or chemical contaminants could not be ascertained. In the absence of additional information about other water quality parameters and about the other cohabiting species, especially given the moderately aggressive temperament of *Polypterus* spp., the roles these may have played remain purely speculative. Live and raw feeds are known to be potential sources of introduction of pathogens, including viruses, into fish populations [35]. The live worms and raw meat that were routinely fed in this case could therefore have served as potential sources of exposure to viral or other inciting pathogens.

The owner initially expressed that he thought the fish had gotten a laceration. However, on examination of the animal, it was determined that as the axial margins of the gular plates became more readily visible as the tumor grew, the owner mistook these margins for the edges of a laceration. Although this mass was not consistent with chronic inflammation, it is known that in domestic animal species such as dogs and cats, chronic inflammation can lead to tumor formation [36, 37]. Chronic inflammation is also considered one of the predisposing factors for cancers in human medicine, particularly when combined with some infectious organisms [38]. Trauma combined with other possible infectious or environmental factors could have caused this tumor in such a young specimen. The definitive underlying cause in this case, however, remains unknown. To the best of our knowledge, this is the first reported case of a tumor in a bichir.

Secondly, careful consideration had to be given to the anaesthetic protocol in this air-breathing species. It was suspected that spiracular air-breathing would be favoured if the animal was to be immersed in an anaesthetic bath and when the animal was removed from the water for surgical excision of the mass. Hence, it was thought that precautionary measures may be necessary to guard against the potential for drowning if using immersion techniques and to overcome the countering effects of aerially acquired oxygen on achieving and maintaining an adequate plane of surgical anaesthesia to facilitate excision of the mass. The potential implications of spiracular air-breathing on anaesthesia induction and maintenance were thus unclear.

On consultation with other aquatic veterinary colleagues, none had prior experience with bichirs and few had experience working with fish that can utilise lungs for respiration. Concerns raised included the variability of responses to injectable anaesthetic agents among fish species, uncertainty regarding the risk of drowning when using an immersion agent such as MS-222, and the dose of MS-222 that would be effective for maintaining the required depth of anaesthesia in this species if the surgical procedure was protracted. Ultimately, it was generally agreed that MS-222 may offer the best response provided the immersion bath used for induction and the anaesthetic water continuously flushed over the gills intraoperatively were either oxygenated or highly aerated to close to oxygen saturation, allowing for maintenance of branchial respiration in preference to spiracular air breathing. Typically, anaesthesia induction with MS-222 is achieved on immersion in concentrations of 100-200 ppm, maintenance of anaesthesia at 50-100 ppm, and sedation at 15-50 ppm [39]. In this case, however, induction and maintenance were achieved at considerably lower concentrations than expected, 50 ppm and 35 ppm, respectively. The duration of the surgical procedure ultimately did not exceed 15 minutes. As such, recovery was smooth and rapid as is typical for short procedures using MS-222. To the best of our knowledge, this report provides the first description of surgical anaesthesia in *Polypterus* spp.

Such cases could further benefit from the availability of liquid nitrogen for cryoablation of the surgical margins to mitigate against the risk of tumor recurrence. Despite the wide excision margins achieved in this case, had the animal gone on to recover further, it would have been monitored for recurrence since this is a likely sequela if all tumor cells were not removed or otherwise destroyed. Another possible consideration for similar cases may be the use of an alternative to meloxicam as part of the pain management regimen. While meloxicam has been used clinically in fish at the dose described, doubt has since been cast on its efficacy [40]. Until more is known, a more widely studied agent in fish, such as morphine, may be of greater benefit to the surgical patient. Given morphine's demonstrated beneficial analgesic effects across a wider range of fish species, its documented pharmacokinetics, and its very few reported side effects across species examined, it may be a reasonable alternative for bichirs [40]. However, interspecies variability in the response to morphine does occur, so caution should always be used when extrapolating responses between species for this or any other agent being considered for pain management [40].

While masses in the gular region of fish are typically assumed to be associated with thyroid hyperplasia (goiter), this case demonstrated that cutaneous neoplasms should not be discounted as possible differentials. Further, surgical planes of anaesthesia can be achieved using tricaine methanesulfonate (MS-222) in *Polypterus* spp. However, we recommend ensuring that the dissolved oxygen in the anaesthetic water is near saturation, exercising caution in each case as the effective dose may be significantly lower than is typical for other species, and supporting the anaesthetised or sedated animal near the water surface when immersed to mitigate the risk of aspiration of water resulting in drowning. Consistent with other teleost species, following short procedures, a smooth, rapid recovery occurs upon immersion in well-aerated fresh water.

## Figures and Tables

**Table 1:** Timeline of the case of gular neoplasia in a saddled bichir (*Polypterus endlicheri endlicheri*).

Day	Presentation/intervention
September 11, 2017	(i) Animal presented for suspected “throat laceration” (ii) Physical examination performed and fine needle aspirate (FNA) scheduled
September 15, 2017	(i) FNA of mass submitted for cytological analysis
September 18, 2017	(i) Cytological analysis revealed evidence of necrosis, deemed nondiagnostic. Reaspiration requested by laboratory
September 22, 2017	(i) FNA repeated and resubmitted for cytological analysis
September 25, 2017	(i) Cytological analysis revealed evidence of necrosis. Biopsy and histological analysis recommended (ii) Surgical excision recommended
October 15, 2017	(i) Animal donated to UWI-SVM by owner
October 30, 2017	(i) Surgical excision and submission of mass for histopathological analysis
November 01, 2017	(i) Animal found dead
November 29, 2017	(i) Histopathological confirmation of cutaneous fibrosarcoma

## Data Availability

Data associated with this report can be made available upon reasonable request.

## References

[1] Zaccone G., Lauriano E. R., Capillo G., Kuciel M. (2018). Air- breathing in fish: air- breathing organs and control of respiration: nerves and neurotransmitters in the air-breathing organs and the skin. *Acta Histochemica*.

[2] Darnet S., Dragalzew A. C., Amaral D. B. (2019). Deep evolutionary origin of limb and fin regeneration. *Proceedings of the National Academy of Sciences of the United States of America*.

[3] Graham J. B., Wegner N. C., Nilsson G. E. (2010). Breathing air in water and in air: the airbreathing fishes. *Respiratory Physiology of Vertebrates: Life with and without Oxygen*.

[4] Graham J. B. (1997). *Air-Breathing Fishes: Evolution, Diversity, and Adaptations*.

[5] Graham J. B., Wegner N. C., Miller L. A. (2014). Spiracular air breathing in polypterid fishes and its implications for aerial respiration in stem tetrapods. *Nature Communications*.

[6] Lechleuthner A., Schumacher U., Negele R. D., Welsch U. (1989). Lungs of *Polypterus* and *Erpetoichthys*. *Journal of Morphology*.

[7] Maina J. N. (1987). The morphology of the lung of the African lungfish, Protopterus aethiopicus, *Protopterus aethiopicus*: A scanning electron-microscopic study. *Cell and Tissue Research*.

[8] Perry S. F., Fernandes M. N., Glass M. L., Kapoor B. G. (2016). Swimbladder-lung homology in basal osteichthyes revisited. *Fish Respiration and the Environment*.

[9] Brainerd E. L., Liem K. F., Samper C. T. (1989). Air ventilation by recoil aspiration in polypterid fishes. *Science*.

[10] Carey J. R., Judge D. S. (2000). *Longevity Records: Life Spans of Mammals, Birds, Amphibians, Reptiles and Fish*.

[11] Kabré G. B., Petter A. J. (1997). Camallanus polypteri n. sp. (Nematoda:Camallanidae) in freshwater fishes from Burkina Faso. *Onderstepoort Journal of Veterinary Research*.

[12] Přikrylová I., Matějusová I., Musilová N., Gelnar M., Harris P. D. (2009). A new gyrodactylid (Monogenea) genus on gray bichir, *Polypterus senegalus* (Polypteridae) from Senegal (West Africa). *Journal of Parasitology*.

[13] Weber E. S. (2010). Geriatric veterinary care for fish patients. *The Veterinary Clinics of North America. Exotic Animal Practice*.

[14] Lackmann A. R., Andrews A. H., Butler M. G., Bielak-Lackmann E. S., Clark M. E. (2019). Bigmouth Buffalo *Ictiobus cyprinellus* sets freshwater teleost record as improved age analysis reveals centenarian longevity. *Communications Biology*.

[15] Noga E. J. (2010). *Fish Diseases: Diagnosis and Treatment*.

[16] Woo P. T. K., Bruno D. (2011). *Fish Diseases and Disorders*.

[17] Coffee L. L., Casey J. W., Bowser P. R. (2013). Pathology of tumors in fish associated with retroviruses: a review. *Veterinary Pathology*.

[18] Grizzle J., Goodwin A., Leatherland A., Woo P. T. K. (1998). Neoplasms and related lesions. *Fish Diseases and Disorders*.

[19] Vergneau-Grosset C., Nadeau M. E., Groff J. M. (2017). Fish Oncology: Diseases, Diagnostics, and Therapeutics. *Veterinary Clinics of North America Exotic Animal Practice*.

[20] Sweet M., Kirkham N., Bendall M., Currey L., Bythell J., Heupel M. (2012). Evidence of melanoma in wild marine fish populations. *PLoS One*.

[21] Groff J. M. (2004). Neoplasia in fishes. *The Veterinary Clinics of North America. Exotic Animal Practice*.

[22] Roberts R. J., Roberts R. J. (2012). Neoplasia of teleosts. *First Pathology*.

[23] Anders K., Hilger I., Moller H. (1991). Lentivirus-like particles in connective tissue tumours of fish from German coastal waters. *Diseases of Aquatic Orgaisms*.

[24] Bowser P. R., Abou-Madi N., Garner M. M. (2005). Fibrosarcoma in yellow perch, Perca flavescens (Mitchill). *Journal of Fish Diseases*.

[25] Dennis M. M., Diggles B. K. (2015). Multicentric orocutaneous fibrosarcoma in a fork-tailed catfish (Hemiarius dioctes). *Journal of Fish Diseases*.

[26] Harshbarger J., Spero P., Wolcott N., Couch J., Fournie J. (2021). Neoplasms in wild fish from marine ecosystems emphasizing environmental interactions. *Pathobiology of Marine and Estuarine Organisms*.

[27] Martineau D., Ferguson H. W., Ferguson H. W. (2006). Neoplasia in fish. *Systemic Pathology of Fish*.

[28] Rezaie A., Dezfuly Z. T., Peyghan R. (2017). Fibrosarcoma in a goldfish (*Carassius auratus*): a case report. *Iranian Journal of Veterinary Science and Technology*.

[29] Luna L. G. (1968). *Manual of Histologic Staining Methods of the Armed Forces Institute of Pathology*.

[30] Hoover K. L. (1984). Hyperplastic thyroid lesions in fish. *National Cancer Institute Monograph*.

[31] Marsh M. C., Vonwiller P. (1916). Thyroid tumor in the sea bass (Serranus). *The Journal of Cancer Research*.

[32] Snieszko S. F., Halver J. (2013). Nutritional fish diseases. *Fish Nutrition*.

[33] Roccabianca P., Schulman F. Y., Avallone G. (2020). *Surgical Pathology of Tumors of Domestic Animals*.

[34] Groff J. M. (2001). Cutaneous biology and diseases of fish. *The Veterinary Clinics of North America. Exotic Animal Practice*.

[35] Yanong R. P. E., University of Florida I. E. (2009). *Fish Health Management Considerations in Recirculating Aquaculture Systems—Part 2: Pathogens*.

[36] Hendrick M. J., Meuten D. J. (2017). Mesenchymal tumors of the skin and soft tissues. *Tumors in domestic animals*.

[37] Munday J. S., Lohr C. V., Kiupel M., Meuten D. J. (2017). Tumors of the alimentary tract. *Tumors in domestic animals*.

[38] Kumar V., Abbas A. K., Aster J. C., Cotran R. S., Robbins S. L., Kumar V., Abbas A. K., Aster J. C., Cotran R. S., Robbins S. L. (2021). Neoplasia. *Robbins & Cotran pathologic basis of disease*.

[39] Lewbart G., Harms C. A. (1999). *Building a dish anesthesia delivery system*.

[40] Chatigny F., Creighton C. M., Stevens E. D. (2018). Updated review of fish analgesia. *Journal of the American Association for Laboratory Animal Science*.

